# Enhanced photoluminescence and photocatalytic properties in Dy-doped sodium zinc molybdate synthesized *via* a green microwave-assisted method†

**DOI:** 10.1039/d5na00047e

**Published:** 2025-04-04

**Authors:** Sourabh Gouraha, Khalid Bin Masood, Sadaf Jamal Gilani, Apoorva Rai, H. S. Tewari, Jai Singh

**Affiliations:** a Department of Pure and Applied Physics, Guru Ghasidas Vishwavidyalaya Bilaspur 495009 India jai.bhu@gmail.com; b Department of Physics, SP College, Cluster University Srinagar J&K 190001 India; c Department of Pharmaceutical Sciences, College of Pharmacy, Princess Nourah bint Abdulrahman University P. O. Box 84428 Riyadh 11671 Saudi Arabia

## Abstract

This study focuses on the structural, photoluminescence and photocatalytic properties of a Dy^3+^-doped sodium zinc molybdate phosphor. The samples were synthesized using a green microwave-assisted method owing to its efficiency in time and energy consumption. The powder X-ray diffraction (P-XRD) analysis confirmed the monoclinic structure of as-synthesized Dy^3+^-doped sodium zinc molybdate with space group *C*2/*m*. The most prominent excitation band appeared at 348 nm under 590 nm, and the emission spectra for all samples exhibited a sharp peak at around 590 nm, which is attributed to the ^4^F_9/2_ → ^6^H_13/2_ electric dipole transition of Dy^3+^ ions. The emission intensity increases with higher Dy^3+^ doping levels, reaching maximum intensity at a concentration of 6 mol% of Dy^3+^ in sodium zinc molybdate. Additionally, the Commission Internationale de l'Éclairage (CIE) chromaticity coordinates of the Dy^3+^ sodium zinc molybdate phosphor places it within the orangish region. Dy^3+^ is also found to play a great role in enhancing the photocatalytic activity of sodium zinc molybdate under UV light. These findings indicate that the as-synthesized phosphor holds promise for white LED and water purification applications.

## Abstract 2

This study focuses on the structural, photoluminescence and photocatalytic properties of a Dy^3+^-doped sodium zinc molybdate phosphor.

## Graphical abstract

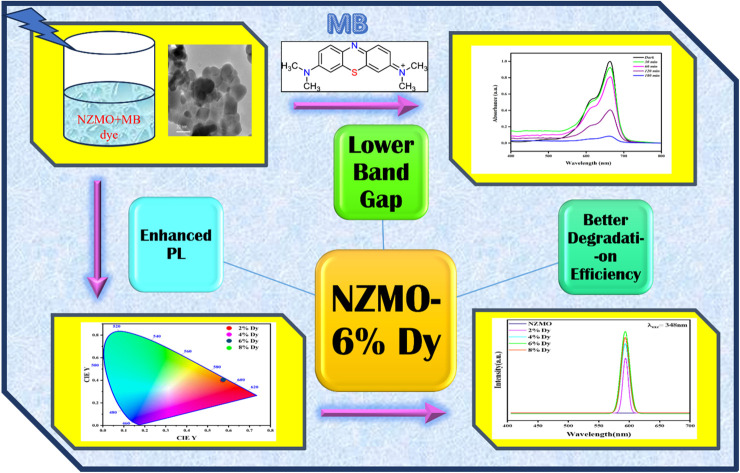

## 1. Introduction

Phosphor materials are essential components in various light emitting display technologies, such as in field emission displays, plasma display panels (PDPs), and lighting applications.^1–3^ White light-emitting diodes (W-LEDs) have emerged as a next-generation light source, offering an alternative to traditional fluorescent lamps due to their benefits, including energy efficiency, quick response time, absence of mercury, low voltage requirements, and environmental friendliness. Currently, conversion-type white LEDs dominate large-scale commercial production worldwide.^4,5^

Fluorescent nanophosphors activated by lanthanides have gained significant attention in diverse applications across areas such as data storage through optical means, field emission display technology, solar energy devices, LED illumination systems, and night vision equipment.^6–8^ These materials exhibit a wide range of photoluminescence (PL) emissions, influenced by transitions such as 4f–4f and 4f–5d. Lanthanide ions (Ln^3+^) are particularly notable for their useful properties, including good biocompatibility, resistance to photo-bleaching, and enhanced photo-stability, making them promising for applications such as cancer cell detection, drug delivery and multimode imaging.^9,10^ However, for efficient PL emission, Ln^3+^ ions require a suitable host matrix that can transfer energy to the ions, enabling the otherwise parity-forbidden 4f–4f transitions through the crystal field effect.^11^ Numerous host materials such as NaLuF_4_, NaYbF_4_, LaF_3_, NaGdF_4_, KGdF_4_, NaScF_4_, and NaYF_4_ have been explored for their potential in bio-imaging applications.^12–14^ These hosts are stable at room temperature under ambient conditions, yet oxides offer additional advantages. Oxide-based hosts, owing to their robust chemical and thermal stability, coupled with their environmentally friendly characteristics, overcome the limitations associated with fluoride hosts.^15,16^ Various oxide hosts, such as molybdates, tungstates, phosphates, gallates, and aluminates, have been explored. In recent years, oxide hosts have garnered particular interest for biomedical applications such as drug delivery, bio-imaging, and diagnosis, owing to their biocompatibility.^17,18^ Red fluorescence, in particular, is highly suited for bio-imaging purposes due to its low scattering and deeper tissue penetration.^13^

The Dy^3+^ ion, with an electron configuration of 4f^9^, exhibits distinctive optical properties.^19^ The transitions of Dy^3+^, specifically ^4^F_9/2_ → ^6^H_15/2_, ^4^F_9/2_ → ^6^H_13/2_, and ^4^F_9/2_ → ^6^H_11/2_, correspond to emissions in the blue, yellow, and red regions of the spectrum, making these transitions significant in the study of white luminescent materials.^20,21^ For instance, Chengaiah and colleagues synthesized ZnMoO_4_ phosphors activated with varying concentrations of Dy^3+^ using a solid-state reaction at 900 °C. Their research revealed a quenching effect of the excited state (^4^F_9/2_) due to energy transfer at higher Dy^3+^ concentrations.^3^ In another study, Yang and team developed Ca_9_Gd(PO_4_)_7_ phosphors, undoped and Dy^3+^-doped. Under ultraviolet excitation at 350 nm, they observed a prominent blue emission, indicating that Dy^3+^ ions were situated in positions with high symmetry and an inversion center.^22^ Similarly, Jamalaiah and colleagues prepared strontium aluminate phosphors with varying Dy^3+^ concentrations. When the Dy^3+^ concentration exceeded 1.0%, electron transitions driven by d–d interactions produced white light, though the phosphor's color rendering index remained low.^23^ As of now, there are very few known reports on Dy^3+^-doped ZnMoO_4_ nanophosphors.

The ZnMoO_4_ host matrix exhibits a crystal structure analogous to that of MgMoO_4_, where the (MoO_4_)^2−^ oxyanion complex serves as the fundamental building block.^24,25^ In this triclinic symmetry wolframite-type structure, molybdenum (Mo) ions are coordinated tetrahedrally by four oxygen (O^2−^) ions. Interestingly, Mo ions occupy three distinct crystallographic sites, which may contribute to the material's unique optical and catalytic properties.^26,27^

ZnMoO_4_ has garnered attention for its versatility in applications such as phosphors, laser crystals, and catalysts due to its robust chemical and thermal stability. The substitution of Zn^2+^ with Dy^3+^ ions introduces additional emission characteristics, making it particularly valuable for luminescent materials. For instance, the combination of yellow and blue emissions from Dy^3+^-doped ZnMoO_4_ results in chromaticity coordinates (*x*, *y*) that fall within the white light region on the CIE diagram. This feature enhances its applicability in white light-emitting devices. In the absence of direct charge compensation, the host matrix compensates by capturing O^2−^ from the air. In the case of ZnMoO_4_:Dy^3+^ phosphors, two Dy^3+^ ions replace three Zn^2+^ ions, and the resulting charge compensation is provided by a zinc vacancy.^28^

In addition to their luminescence properties, rare-earth-doped luminescent materials have shown significant promise in photocatalytic applications. Dy^3+^-doped metal oxides, such as ZnWO_4_,^29^ exhibit excellent photocatalytic properties due to their enhanced light absorption and efficient charge carrier separation. These materials harness the energy from UV and visible light to degrade organic pollutants, including dyes and phenolic compounds, through hydroxyl and superoxide radical generation. Dy^3+^ ions, with their unique 4f^9^ configuration, also facilitate electron trapping, reducing recombination rates and boosting photocatalytic efficiency. Studies have highlighted their effectiveness in environmental remediation, particularly for wastewater treatment, by demonstrating substantial dye degradation efficiencies. The incorporation of Dy^3+^ into oxide hosts not only improves luminescence but also tailors band gap properties, making these materials versatile for dual applications in optoelectronics and photocatalysis.^30,31^

Furthermore, several methods have been designed for the development of phosphors, including solid-state reaction,^32^ co-precipitation,^33^ the Pechini method,^34^ pulsed laser deposition method,^35^ sol–gel techniques,^36^ combustion synthesis,^37^ organic gel-thermal decomposition,^38^ hydrothermal,^39^ and solvothermal synthesis.^40^ Among various synthesis approaches, the microwave-assisted method is particularly noteworthy due to its efficiency in time and energy consumption. This technique leverages microwave irradiation to rapidly heat the reactants, promoting uniform nucleation and accelerating reaction rates. Unlike traditional heating methods, microwave energy directly interacts with the material at a molecular level, enabling faster reaction times and minimizing energy wastage. This leads to reduced processing period and enhanced control over particle size and morphology, which are critical for phosphor properties. Consequently, microwave-assisted synthesis is highly advantageous for producing high-quality phosphors with consistent and desirable characteristics, making it an excellent choice for applications where precision and resource efficiency are key.^41,42^

## 2. Experimental details

### 2.1. Synthesis method

In brief, zinc nitrate hexahydrate (10 mmol) and sodium molybdate dihydrate (10 mmol) were each made to dissolve in 20 ml of deionized water and stirred at room temperature for 30 minutes. The obtained mixture was then put in sealed glass vessels with a safety valve and heated in a 100 W microwave (2.45 GHz) for 15 minutes. Afterward, the system was naturally cooled to room temperature. The final product was washed thrice with water and ethanol, then dried at 120 °C for 12 h.

### 2.2. Characterization techniques

The synthesized material was thoroughly characterized using several experimental techniques. The phase formation and crystallinity of the nanophosphors were examined using X-ray diffraction (XRD), collected with a Rigaku Mini Flex 600 diffractometer employing CuKα radiation (*λ* = 1.54046 Å), across a 2*θ* range of 10° to 52°. The Raman spectra were observed using Technos Japan Raman spectrometer. The microstructural and morphological properties were assessed using scanning electron microscopy (Zeiss EVO-10) and high-resolution transmission electron microscopy (HR-TEM, JEM 2100, Joel, operating at 200 kV). Fourier Transform Infrared (FTIR) spectroscopy (PerkinElmer, 1600) was conducted in the wavenumber range of 4000–400 cm^−1^. UV-Vis absorption spectra were recorded on an Analytical Technology UV-Vis Spectrophotometer within the 200–550 nm range.

### 2.3. Photocatalytic experimental protocol

For dye degradation study of methylene blue (MB), a solution of MB was prepared using 2 mg MB in 200 ml of distilled water. The solution was constantly stirred at room temperature in the dark for an hour. Later on, a 120 W halogen lamp was used to expose the solution to a visible light source. Separately, 15 mg of catalyst was added to 30 ml of MB solution using continuous stirring under visible light. To capture the UV spectrum, the solution was separately taken in a cuvette at various time intervals, which indicates the measurement of the dye's photodegradation.

## 3. Results and discussion

### 3.1. XRD analysis

**Fig. 1:**
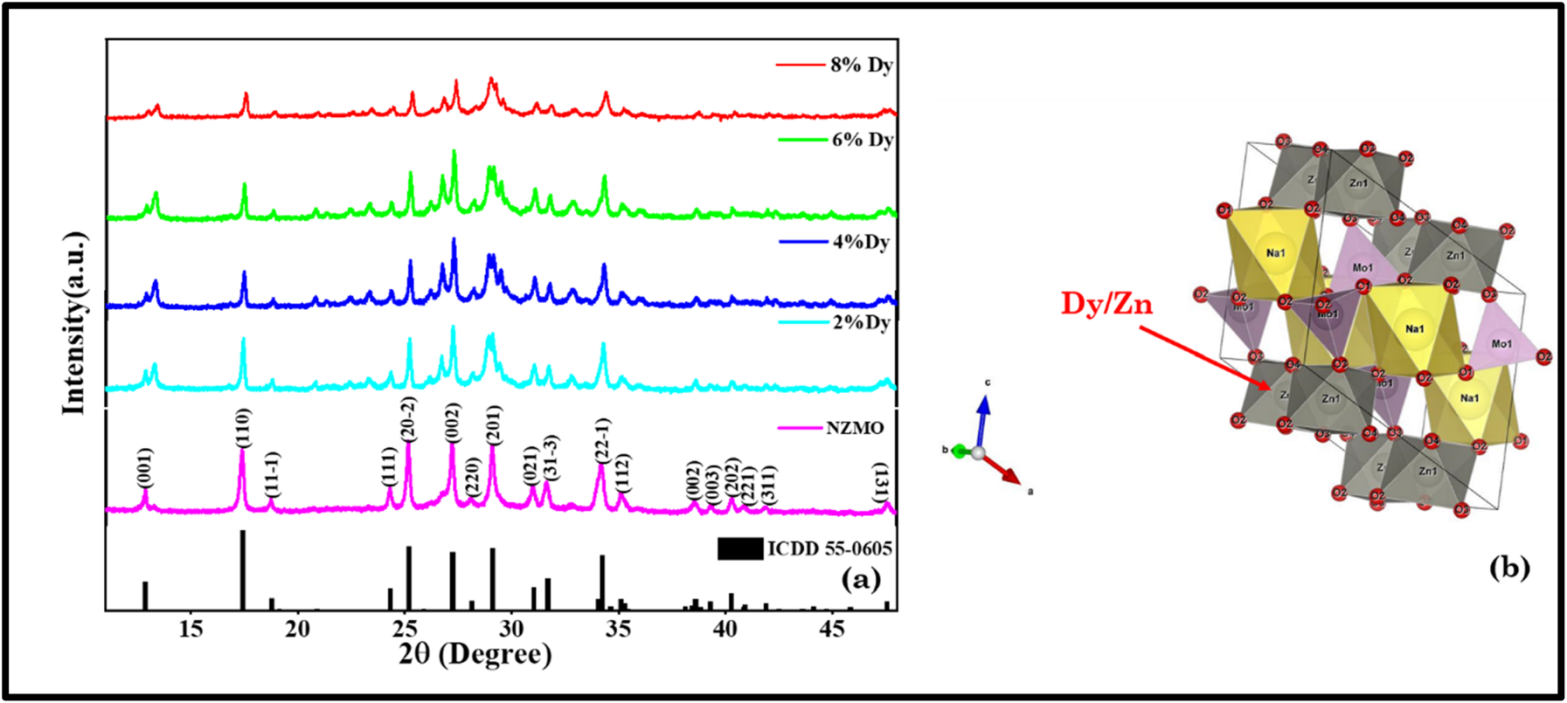
(a) XRD spectra of as synthesised NZMO nanophosphors with varying Dy^3+^ concentrations and (b) polyhedral representation of Dy-doped NZMO.

Fig. 1(a) presents the XRD patterns of un-doped and Dy^3+^-doped NZMO phosphors, which matches well with ICDD card for Na(OH)Zn_2_(MoO_4_)·2.5H_2_O. The observed peak positions align well with the standard NZMO, confirming its monoclinic structure with space group *C*2/*m*. No extra peaks are detected in the diffraction patterns. The average crystallite sizes were determined using the Scherrer equation, which was applied to the full width at half maximum (FWHM) of the primary diffraction peak.^43^
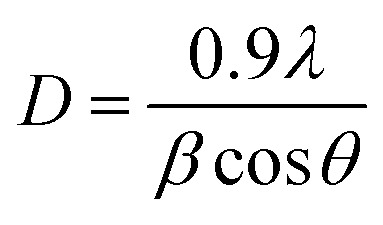
where *β* is the broadening of the diffraction line measured at half maximum intensity (radians) and *λ* = 1.5406 Å is the wavelength of CuK_α_. The calculated crystallite sizes, ranging from 15 to 30 nm, are consistent with the broad diffraction peaks observed, thereby validating the accuracy of the method used.^44^

The absence of diffraction peaks related to Dy^3+^ in the XRD pattern of doped NZMO is confirmed by the other studies. Fig. 1(b) provides a schematic polyhedral of the monoclinic phase of NZMO, created using VESTA Software. This model highlights a high degree of inversion symmetry within the structure. As shown, each Zn^2+^ ion is bonded with six oxygen atoms, forming [ZnO_6_] octahedral units. Mo^6+^ ions, alternatively, coordinate with four oxygen atoms, resulting in [MoO_4_] tetrahedral units. The structure also reveals that Na^+^ ions are surrounded by six oxygen atoms. Within the unit cell, oxygen atoms bridge connections between Zn atoms as well as between Na and Mo atoms. The layered arrangement comprises 8 zinc, 4 sodium, and 10 molybdenum atoms per unit cell. The consistent XRD patterns across doping concentrations, despite the introduction of Dy^3+^, align with the findings of Jain *et al.*,^16^ who demonstrated that trivalent Eu^3+^ ions substitute Zn^2+^ in the same NZMO host lattice, with the lattice accommodating the substitution through minor adjustments and charge compensation mechanisms such as Zn^2+^ vacancies or interstitial defects. Although Dy^3+^ (ionic radius: 0.912 Å for coordination number 6) is larger than Zn^2+^ (0.74 Å), the NZMO lattice exhibits sufficient flexibility to accommodate this mismatch. Studies on similar molybdate systems, such as Eu^3+^-doped ZnMoO_4_,^15^ show that trivalent lanthanides can occupy Zn^2+^ octahedral sites despite size differences, with local distortions and charge-balancing defects stabilizing the structure. In our case, the microwave-assisted synthesis method likely enhances defect formation, facilitating the incorporation of Dy^3+^ at Zn^2+^ sites. The consistent monoclinic phase across doping levels further supports this adaptability, suggesting that Dy^3+^ replaces Zn^2+^ with minimal lattice strain.

### 3.2. FTIR study

**Fig. 2:**
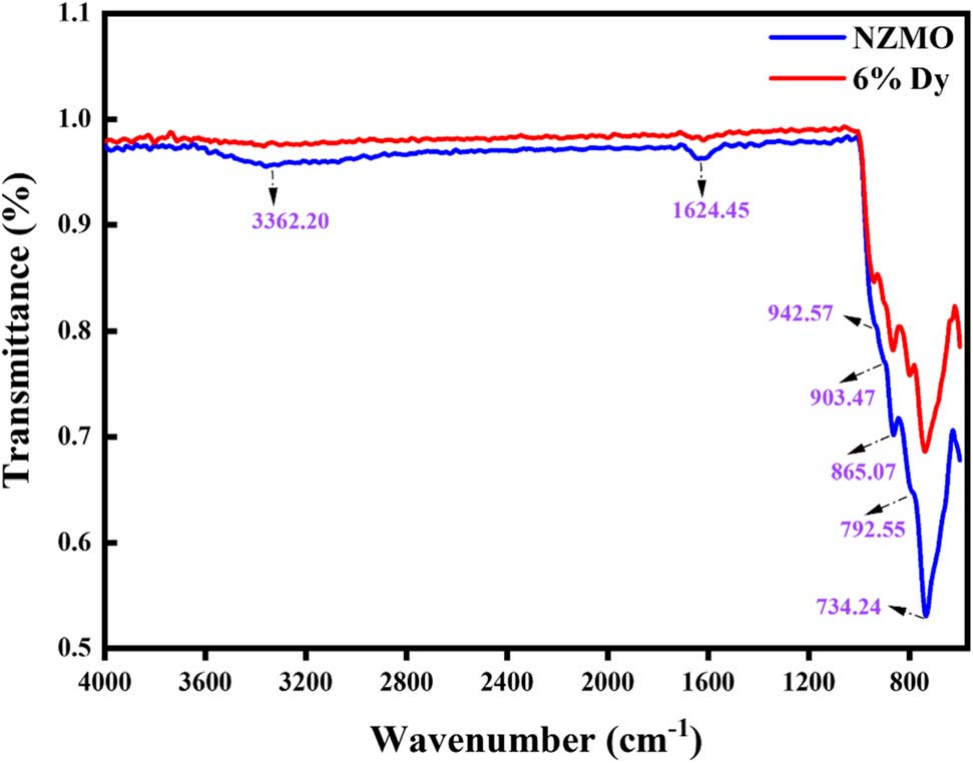
FTIR spectra of NZMO and 6 mol% Dy^3+^-doped NZMO.

Fig. 2 illustrates the FTIR spectra for pure NZMO and 6% Dy-doped NZMO phosphors. It is noted that, despite increasing concentrations of Dy^3+^ ions, the peaks show no positional change of the FTIR spectra associated with NZMO. This suggests that the host lattice remains structurally stable for the different Dy^3+^ concentrations. The bands observed in the 700–1000 cm^−1^ range are primarily associated with the bending and stretching vibrational modes of Zn–O–Mo.^45,46^ Meanwhile, the band around 870 cm^−1^ arises from the bending and asymmetric stretching vibrations of Mo–O.^45^ Additionally, the bands at 1624 cm^−1^ and 3362 cm^−1^ are attributed to H–O–H bending and O–H stretching vibrations, respectively.^47–49^

### 3.3. Raman study

**Fig. 3:**
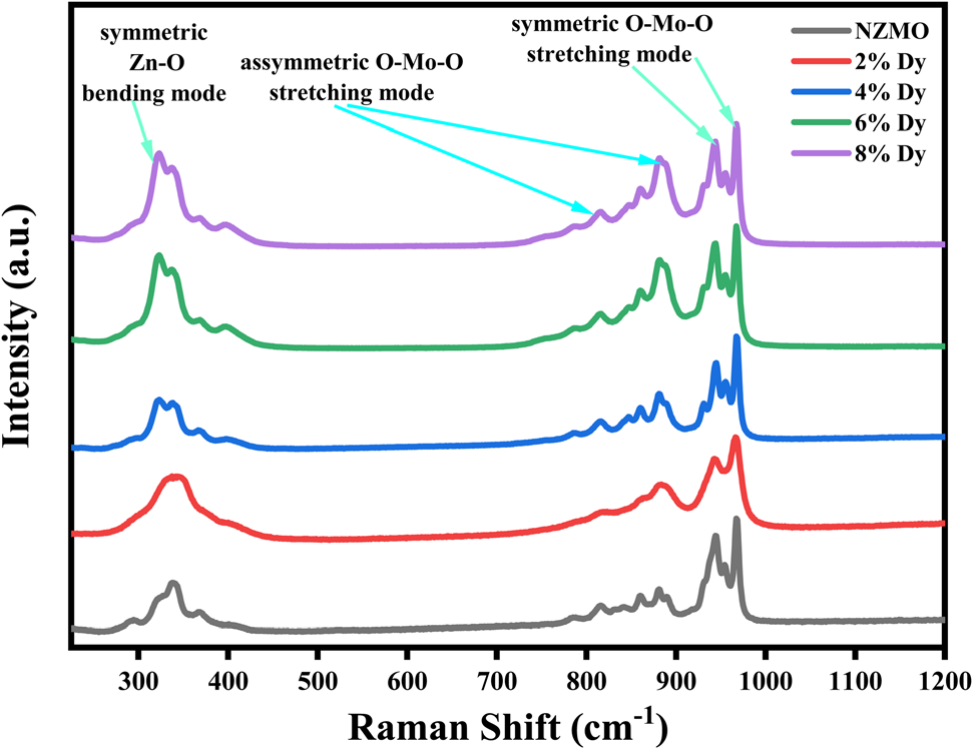
Raman spectra of as synthesised NZMO nanophosphors with varying Dy^3+^ concentrations.

The Raman spectra of the prepared samples are depicted in Fig. 3. The observed bands are attributed to the internal and external vibrations of the Mo–O unit within the host lattice. Bands ranging from 300 to 1000 cm^−1^ are due to internal vibrations of the [MoO_4_] polyhedra, while those below 200 cm^−1^ are linked with external vibrations of the host material. The prominent bands identified in this study occur at approximately 968, 955, 944, 930, 888, 880, 860, 846, 815, 786, 520, 337 and 322 cm^−1^. The bands within the 880-950 cm^−1^ range correspond to the symmetric stretching mode (υ_1_) of Mo–O, and those in the 750–880 cm^−1^ range correspond to the antisymmetric stretching mode (υ_3_) of the [MoO_4_]^2−^ tetrahedral unit.^49,50^ The bands around 320 and 350 cm^−1^ represent the symmetric (υ_2_) bending mode associated with the Mo–O bond.^15,51^ These Raman data align with the phase transition study of ZnMoO_4_.^49,50^ Notably, the intensity of the ∼520 cm^−1^ band in Tb^3+^-doped ZnMoO_4_ is lower compared to that in Eu^3+^-doped ZnMoO_4_, likely due to some distortions in the Mo–O bond.^52,53^

### 3.4. HR-TEM analysis

**Fig. 4:**
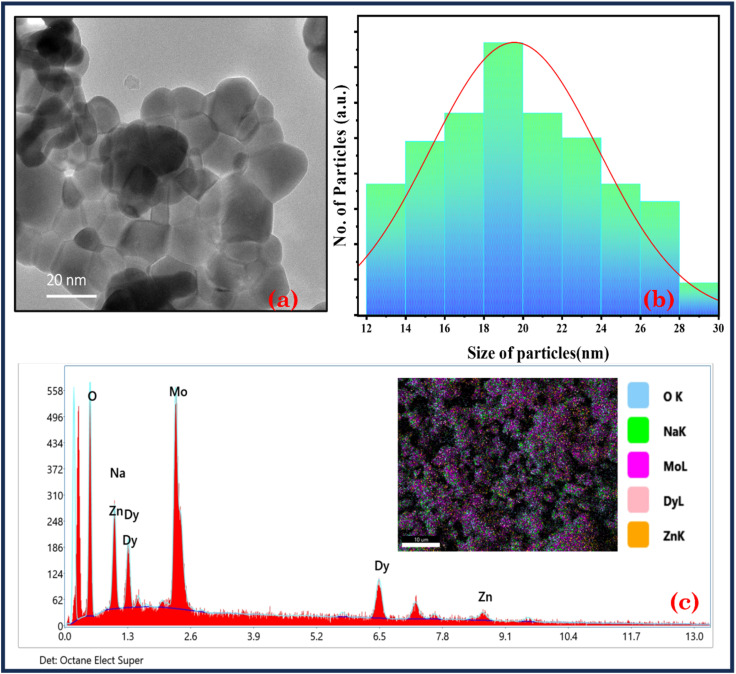
(a) HR-TEM image of NZMO:6 mol% Dy^3+^, (b) particle size histogram of NZMO:6 mol% Dy^3+^ and (c) EDX spectra of NZMO:6 mol% Dy^3+^; inset of (c) elemental mapping image of NZMO:6 mol% Dy^3+^.

Fig. 4(a) shows the HR-TEM analysis of the NZMO:6 mol% Dy^3+^ sample, which displays a variety of spherical and rectangular particle shapes, highlighting the material's morphological diversity. The micrograph also indicates that most particles are in the range of 18–20 nm in size, revealing a consistent nanostructure. This particle size range is corroborated by the particle size distribution observed from histogram measurements (Fig. 4(b)), confirming uniformity across the sample. The EDX and elemental mapping (Fig. 4(c) and inset of 4(c)) further confirm the presence of all targeted elements—sodium, zinc, molybdenum, oxygen, and dysprosium within the synthesised material. Elemental mapping specifically demonstrates a uniform spatial distribution, evenly dispersing throughout the sample. This homogeneity indicates successful doping and synthesis, producing a material with a stable structure and consistent composition. These combined results validate the effectiveness of the synthesis process.

### 3.5. UV-visible spectroscopy

**Fig. 5:**
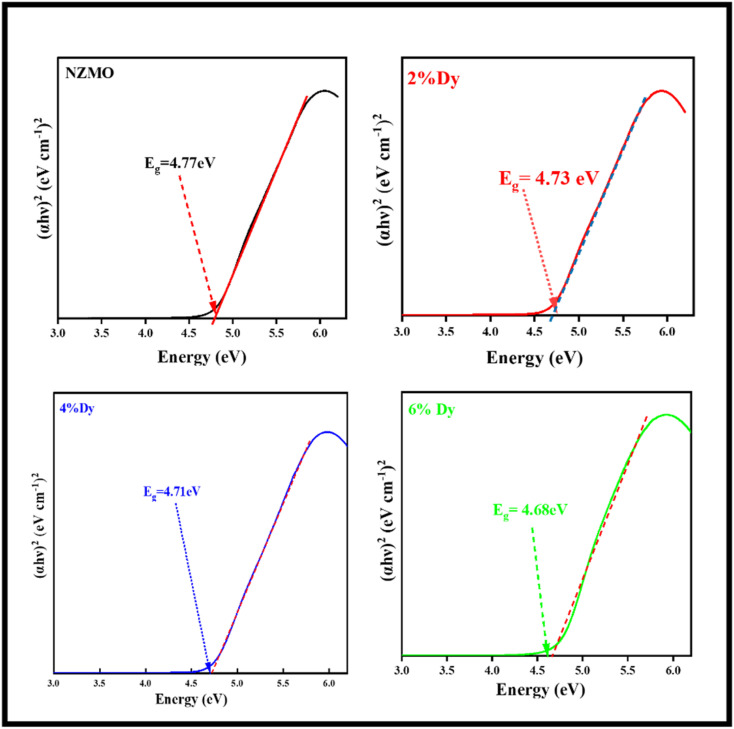
UV-Vis spectra of as synthesised NZMO nanophosphors with varying Dy^3+^ concentrations with band gaps.

The UV-Visible absorbance spectra of the as prepared samples are presented in Fig. S1.† The spectra show a prominent absorption peak in the ultraviolet region, which is ascribed to the electron transfer occurring from the oxygen ligands to the molybdenum atoms in the [MoO_4_]^2−^ complex. In the case of doped samples, no significant shifting in the absorption peaks related to the host material is observed. However, in the doped samples, the absorbance shifts to a longer wavelength with increasing concentrations of Dy^3+^. However, the incorporation of Dy^3+^ ions does cause slight alterations to the band structure of NZMO, affecting the number of photons absorbed by the phosphor.^54^ This is evident from the absorption spectra, where the intensity of the absorption differs between the doped samples. The optical band gap of ZnMoO_4_ was determined utilising Wood and Tauc's method,^55,56^ which gives an empirical equation to determine the optical band gap:

1 *αhv* = *K*(*hν* − *E*_g_)^*n*^

where α represents the absorbance, *h* is Planck's constant, *ν* denotes the frequency, and *E*_g_ refers to the optical band gap. The exponent *n* takes specific values depending on the nature of the electronic transition: 1/2 for direct allowed, 2 for indirect allowed, 3/2 for direct forbidden, and 3 for indirect forbidden transitions. Since α-ZnMoO_4_ exhibits a direct band gap, the optical band gap is calculated using the (*αhν*)^2^*vs.* photon energy plot, which is suitable for direct transitions. The band gap results for the prepared samples are shown in Fig. 5. The estimated band gap values, derived using eqn (1) are approximately 4.77, 4.73, 4.71, and 4.68 eV, which are close to the known band gap of β-ZnMoO_4_.^16^ A shift in the band gap is observed, likely due to the introduction of defects into the crystal structure through Dy^3+^ doping.

### 3.6. Photoluminescence study

**Fig. 6:**
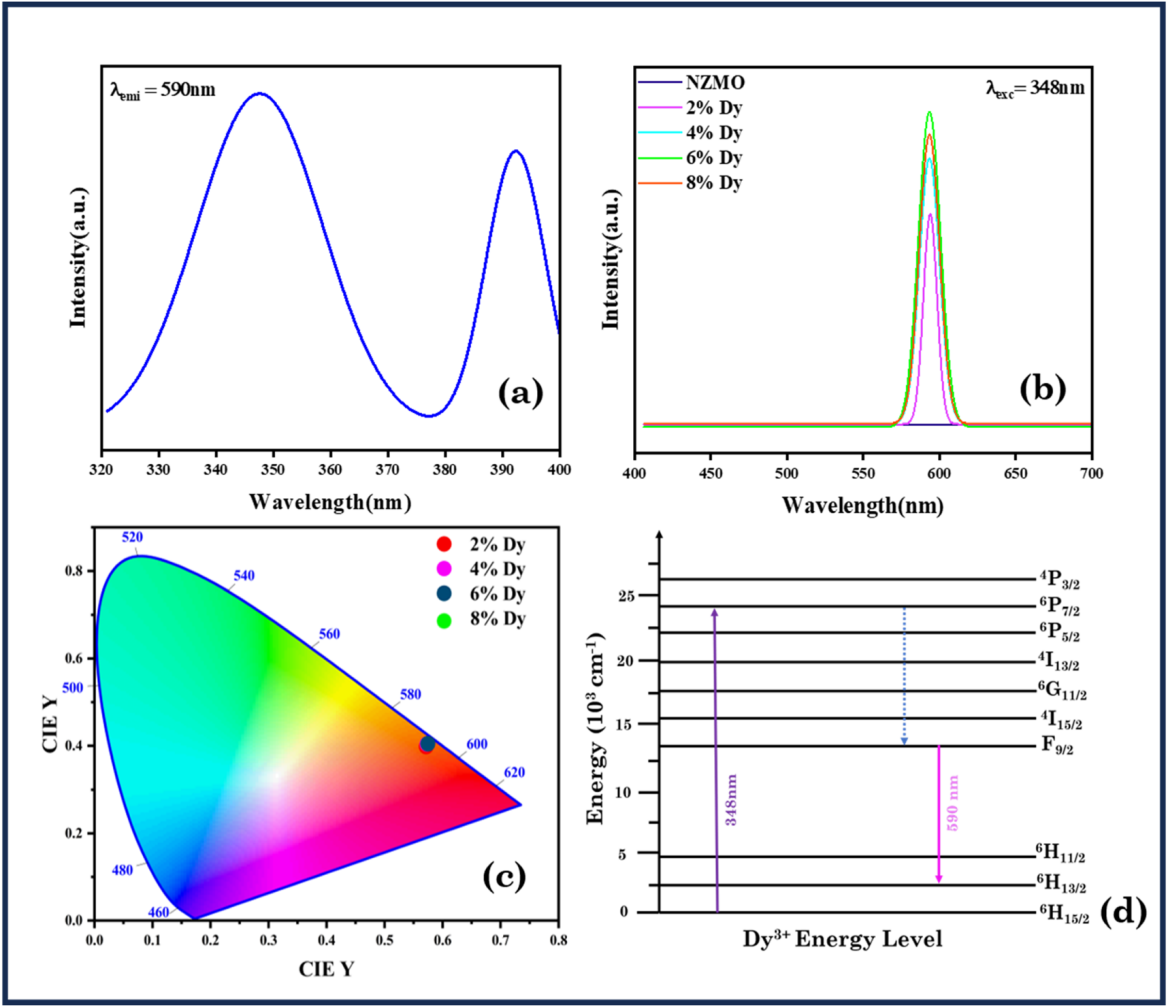
(a) Excitation spectra of NZMO:6 mol% Dy^3+^, (b) emission spectra of NZMO nanophosphors with varying Dy^3+^ concentrations, (c) CIE chromaticity diagram of NZMO nanophosphors with varying Dy^3+^ concentrations and (d) energy level diagram of Dy^3+^.

Fig. 6 illustrates the photoluminescence (PL) excitation and emission spectra CIE chromaticity diagram as well as the energy level diagram for the synthesized NZMO:Dy^3+^ phosphor. In Fig. 6(a) the excitation spectrum displays distinct bands at 348 and 393 nm, corresponding to the ^6^H_13/2_ → ^6^P_7/2_ and ^6^H_13/2_ → ^4^I_13/2_ of Dy^3+^ ions, respectively. The most prominent excitation band appears at 348 nm under 590 nm, prompting the emission spectra to be recorded under this excitation wavelength. The emission spectra for all samples exhibit a sharp peak at around 590 nm, attributed to the ^4^F_9/2_ → ^6^H_13/2_ electric dipole transition of Dy^3+^ ions. Although the emission band's profile remains consistent across different Dy^3+^ concentrations, its intensity increases with higher Dy^3+^ doping levels, as seen in Fig. 6(b), reaching maximum intensity at a concentration of 6 mol% before a drop at 8 mol% due to quenching since as the Dy^3+^ concentration increases, the average distance between ions decreases, enhancing interactions such as electric dipole–dipole coupling according to Dexter's theory; this multipolar interaction facilitates energy migration among Dy^3+^ ions, potentially channeling energy to quenching centers, such as lattice defects, impurities, or surface states where it is dissipated non-radiatively.^57^ The CIE chromaticity coordinates of the synthesized phosphor, shown in Fig. 6(c), place it within the orangish region, These PL findings indicate that the synthesized phosphor holds promise for white LED applications. A diagram of the potential energy transitions associated with each observed emission is provided in Fig. 6(d).

### 3.7. Photocatalytic study

Utilising Methylene Blue (MB) dye, the photocatalytic activity of 6% Dy-doped NZMO was examined under visible light. To decolorise the dye molecules, MB measures the reaction in the presence of Dy-doped NZMO under the exposure to visible light at regular time intervals. Beer-Lambert law aids in defining the connection between the precise amount of a chemical present and its absorbance of UV-visible light. In the case of MB, UV-Visible absorption spectra show a major absorption band at around 664 nm.

**Fig. 7:**
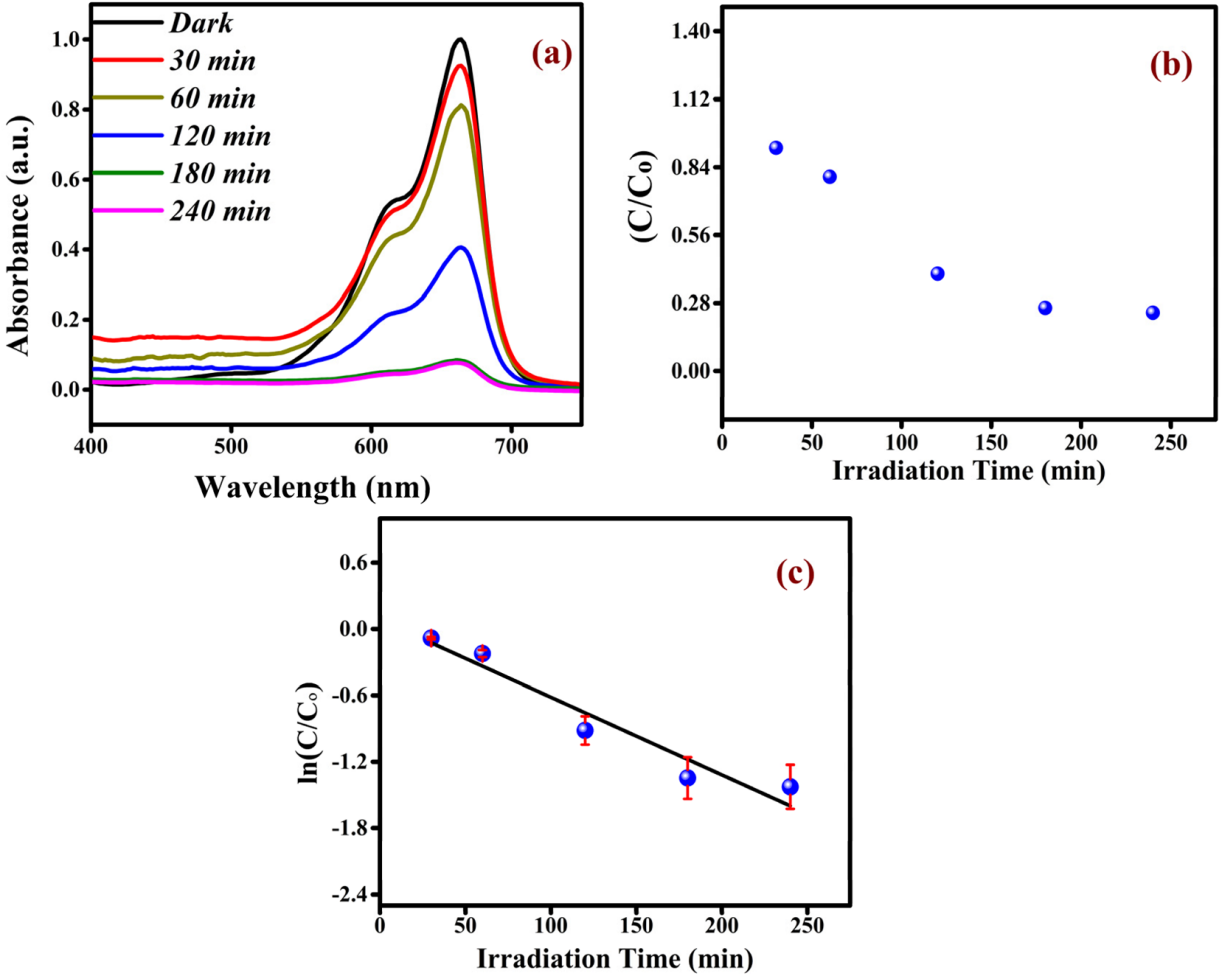
(a) Photocatalytic dye degradation spectrum of MB solution by Dy-doped NZMO catalyst. (b) Degradation efficiency and (c) first order kinetics of photodegraded MB.

Fig. 7(a) shows the photodegradation spectra of MB by 6% Dy-doped NZMO. The degradation efficiency is calculated using the following equation:Degradation efficiency (%) = (*C* − *C*_0_)/*C* × 100where *C* is the initial concentration of MB dye and *C*_0_ is the concentration of MB at *t* min.

**Fig. 8:**
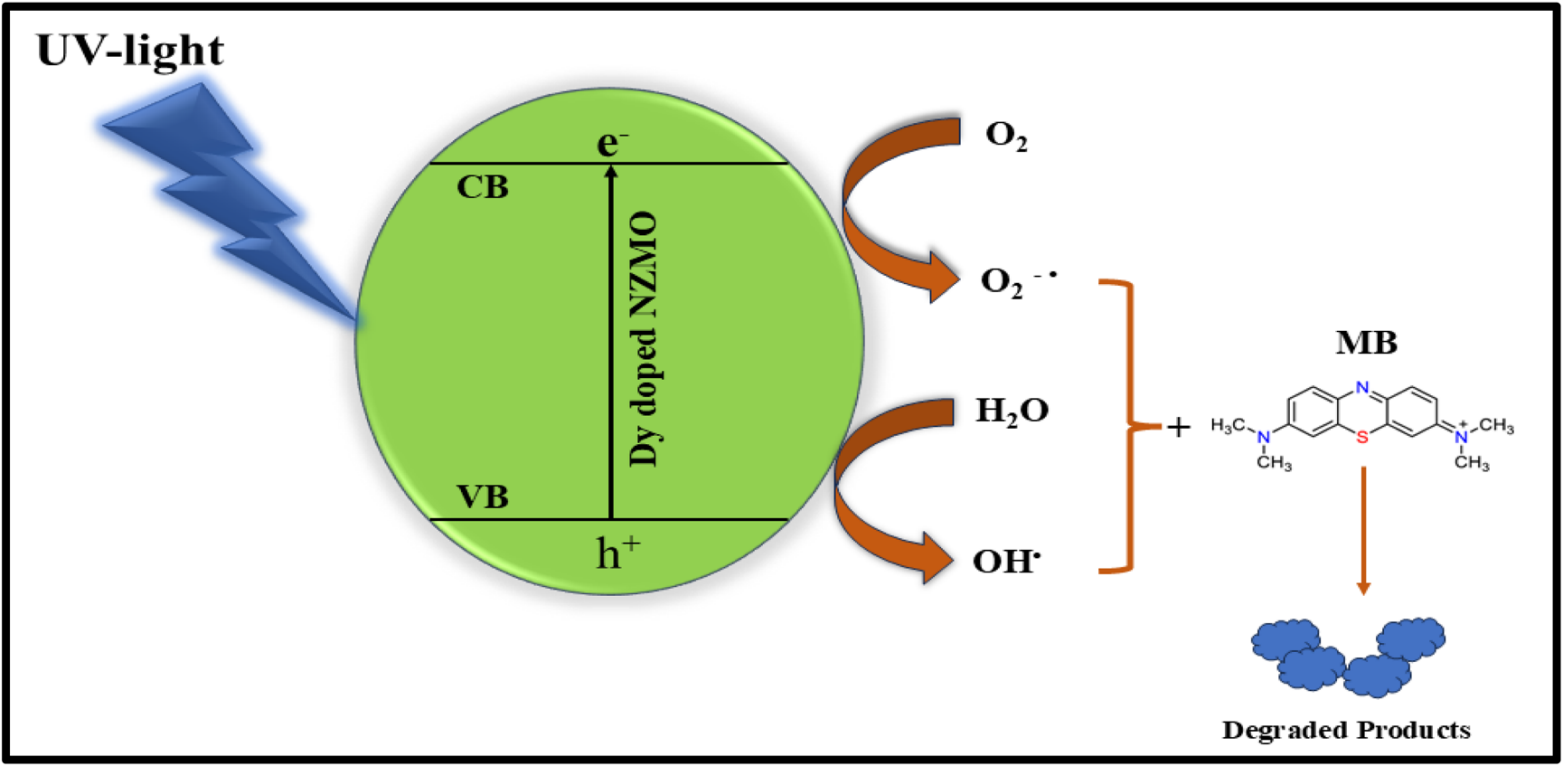
Schematic of photocatalysis mechanism.

It is evident from Fig. 7(a) that the degradation efficiency is 88% in 240 min for the Dy-doped NZMO material, which is higher than undoped NZMO. The degradation of methylene blue dye through photodegradation generally follows first-order reaction kinetics, where the rate constants indicate the efficiency of degradation. Fig. 7(b) demonstrates the exponential decline of the concentration over its initial value with respect to irradiation time for NZMO doped with dysprosium. To evaluate the rate constant for the reaction, a graph is plotted between ln (*C*_0_/*C*_*t*_) and irradiation time as shown in Fig. 7(c). This graph corroborates the pseudo first-order chemical kinetics and the linear fit, where the slope of the straight line represents the rate constant for the reaction. Alternately, some complexes degraded more slowly than expected, with the concentration taking longer to fall to half the initial amount, resulting in a lower observed rate constant. Overall, the results highlight the doping effect on the photocatalytic efficiency and kinetics of degradation. To further oxidise organic dyes, negative species such as OH or organic pollutants can readily trap photoinduced holes, whereas electron acceptors such as adsorbed O_2_ can easily catch photoelectrons. This is because the oxygen vacancies act as electron captures to limit e and h recombination. Furthermore, semiconducting materials' surfaces produce oxygen vacancies as active species, which aid in the photodegradation of organic dyes. The following formulas can be used to describe the relevant photocatalytic reaction process as depicted in Fig. 8.Dy-NZMO + hν → e^−^ + h^+^O_2_ + e^−^ → O_2_˙^−^OH^−^ + h^+^ → OH˙OH˙/O_2_˙^−^ + MB → decomposed product

Dysprosium can exist as Dy^3+^ and Dy^4+^ ions, allowing it to play a greater role in enhancing the photocatalytic activity of NZMO under UV light. When exposed to light, Dy^3+^ readily donates an electron to molecular oxygen adsorbed on the surface, converting to Dy^4+^ and facilitating the reduction of O_2_ to superoxide. This photoinduced charge transfer sequence amplifies the reaction rate compared to pure NZMO. In contrast, photogenerated electrons in the conduction band are capable of transforming Dy^4+^ back to Dy^3+^. The intricate interplay between the shifting oxidation states of dopants, impacted by incoming photons and mobile charges, underlies how dysprosium doping amplifies NZMO's photoactivity. Its absorption edge within the ultraviolet spectrum, unaltered from the bare semiconductor, facilitates the stimulation and subsequent reaction. Ultimately, the rare earth element dysprosium is able to decelerate the recombination of light-generated electron–hole pair and optimize interfacing charge conveyance. Consequently, the mechanization refines the photocatalytic activity of Dy-doped NZMO.^58–60^

**Fig. 9:**
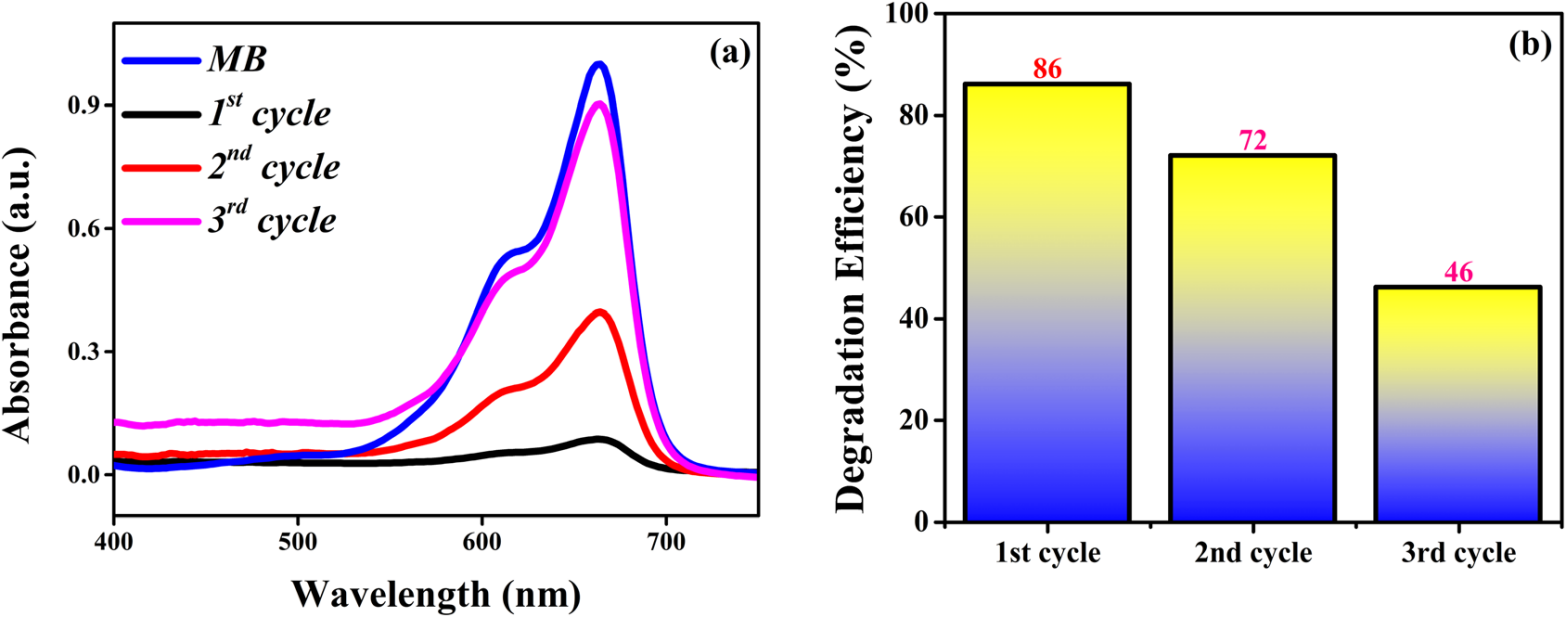
(a) Photodegradation spectra and (b) degradation efficiency of MB using doped NZMO after three reusability cycles.

Furthermore, to assess the stability and reusability of the NZMO catalyst, a comprehensive investigation was conducted. The catalyst was initially employed for the degradation of MB in a reaction mixture. Following the completion of the first cycle, the catalyst was carefully separated from the reaction mixture using centrifugation. The recovered catalyst was thoroughly washed several times with deionized water to remove any residual reactants or byproducts. Subsequently, the washed catalyst was dried in a vacuum oven to remove any moisture and prevent degradation. The dried NZMO catalyst was then reused for the second and third cycles consecutively for the degradation of MB dye, following the same reaction conditions (centrifugation, washing and drying) as the first cycle.^61^ The results of the three consecutive cycles revealed a gradual decline in the catalyst's efficiency. The percentage degradation of MB was found to be 86% in the first cycle, 72% in the second cycle, and 46% in the third cycle as depicted in Fig. 9(a and b). This decrease in catalytic activity corresponds to a reduction in efficiency from 86% to 46% over the three cycles.

**Fig. 10:**
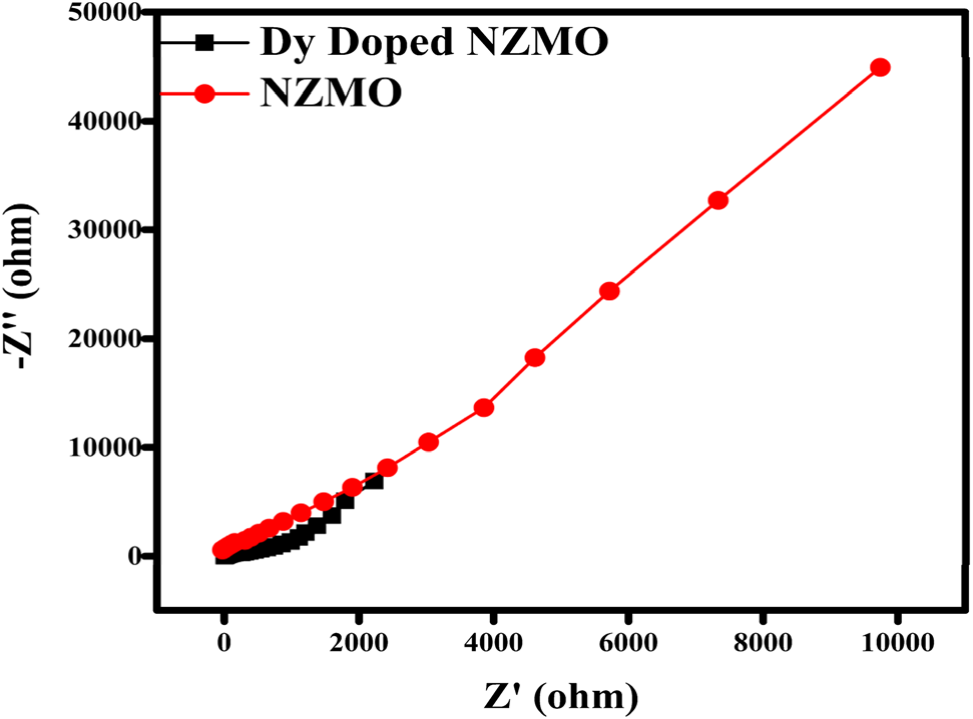
EIS spectrum of NZMO and Dy-doped NZMO.

The observed decline in catalytic performance can be attributed to several factors. One possible reason is the loss of catalyst particles during the washing process, which reduces the overall amount of active catalyst available for the reaction. Additionally, the repeated use of the catalyst may lead to a reduction in active sites, decreasing its ability to facilitate the reaction. Leaching of metal ions from the catalyst and agglomeration of particles may also contribute to the decreased catalytic activity as also reported by Hussain *et al.*^62^ Furthermore, the Nyquist arc radius, a crucial characteristic for assessing charge transport, was revealed through the electrochemical impedance spectroscopy (EIS) analysis, which was carried out from 1 Hz to 10 000 Hz frequency range as shown in Fig. 10. In general, information regarding resistance and interfacial charge migration can be obtained from the semicircle's radius in Nyquist plots; a smaller radius indicates a better charge flow or lower interfacial resistance than larger radii.^63,64^ High conductivity, efficient charge separation, and the fastest interfacial charge transfer were provided by Dy-doped NZMO. The Dy-doped NZMO sample has a lower arc radius than NZMO as shown in Fig. 10, which suggests a considerable drop in the electronic recombination rate and a faster transfer rate of photogenerated charge carriers as also reported by Nicácio *et al.*^65^ These findings demonstrate that Dy doping effectively raised the sample's photocatalytic activity.

The reduced charge transfer resistance and increased photocurrent intensity imply that Dy adds advantageous intermediate states to the band gap, promoting effective charge transport and lowering recombination losses. Dy doping, as opposed to the undoped sample, is therefore a useful tactic to enhance the material's photocatalytic qualities.

## 4. Conclusion

In summary, monoclinic sodium zinc molybdate phosphor was successfully synthesized *via* microwave-assisted synthesis method. The tetrahedral configuration of MoO_4_^2−^ was confirmed by Raman, FTIR and UV studies. The estimated band gap values are found to be 4.77, 4.73, 4.71, and 4.68 eV for undoped and 2%, 4% and 6% Dy-doped NZMO samples, respectively. The excitation spectrum of the as prepared samples displayed distinct bands at 348 and 393 nm, corresponding to the ^6^H_13/2_ → ^6^P_7/2_ and ^6^H_13/2_ → ^4^I_13/2_ transitions of Dy^3+^ ions, respectively, with the most prominent excitation band appearing at 348 nm under 590 nm. The emission spectra for all the samples exhibit a sharp peak at around 590 nm, attributed to the ^4^F_9/2_ → ^6^H_13/2_ electric dipole transition of Dy^3+^ ions. Additionally, Dy^3+^ doping enhances NZMO's photocatalytic activity, achieving 88% methylene blue (MB) degradation in 240 minutes for the Dy^3+^-doped NZMO sample. Reusability tests over three cycles (86% to 46% efficiency) and EIS measurements further reveal Dy^3+^'s role in reducing charge transfer resistance, supporting its photocatalytic efficacy.

## Data availability

Data will be made available upon proper request.

## Conflicts of interest

There are no conflicts to declare.

## Supplementary Material

NA-007-D5NA00047E-s001
