# Supplementary material for: Enhanced photoluminescence and photocatalytic properties in Dy-doped sodium zinc molybdate synthesized via a green microwave-assisted method
Source: Nanoscale Adv. 2025 Apr 4;7(10):3038–48. doi: 10.1039/d5na00047e (PMC11969378; doi:10.1039/d5na00047e)
Supplement: NA-007-D5NA00047E-s001 [file NA-007-D5NA00047E-s001.pdf]

Supplementary Information (SI) for Nanoscale Advances.
This journal is © The Royal Society of Chemistry 2025

Supplementary information

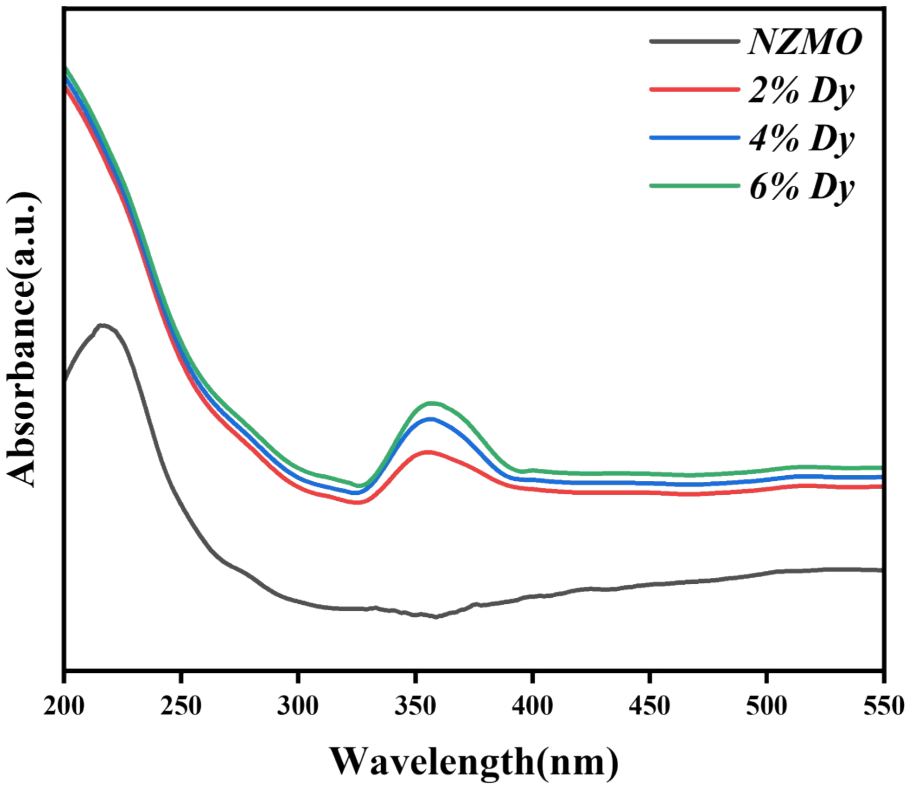

Figure S1 UV-Vis absorption spectra of NZMO with different Dy doping concentartions
